# Cardiac Autonomic Dysfunction and Incidence of *de novo* Atrial Fibrillation: Heart Rate Variability vs. Heart Rate Complexity

**DOI:** 10.3389/fphys.2020.596844

**Published:** 2020-12-08

**Authors:** Niels Wessel, Karsten Berg, Jan F. Kraemer, Andrej Gapelyuk, Katrin Rietsch, Tino Hauser, Jürgen Kurths, Dave Wenzel, Norbert Klein, Christof Kolb, Roberto Belke, Alexander Schirdewan, Stefan Kääb

**Affiliations:** ^1^Department of Physics, Humboldt-Universität zu Berlin, Berlin, Germany; ^2^BIOTRONIK, Berlin, Germany; ^3^Potsdam Institute for Climate Impact Research, Potsdam, Germany; ^4^Centre for Analysis of Complex Systems, Sechenov First Moscow State Medical University, Moscow, Russia; ^5^Clinic for Cardiology and Angiology, University Hospital Magdeburg, Magdeburg, Germany; ^6^St. Georg Hospital, Leipzig, Germany; ^7^Deutsches Herzzentrum München, Klinik für Herz- und Kreislauferkrankungen, Abteilung für Elektrophysiologie, Faculty of Medicine, Technische Universität München, Munich, Germany; ^8^Sana Klinikum Lichtenberg, Berlin, Germany; ^9^Medical Center of Ludwig-Maximilians-University of Munich, Munich, Germany

**Keywords:** heart rate variability, heart rate complexity, cardiac autonomic dysfunction, atrial fibrillation, implantable cardioverter defibrillator

## Abstract

**Background:**

The REACT DX registry evaluates standard therapies to episodes of long-lasting atrial tachyarrhythmias and assesses the quality of sensing and stability of the lead and the implantable cardioverter-defibrillator (ICD) (BIOTRONIK Lumax VR-T DX and successors) over at least a 1-year follow-up period.

**Objective:**

To study the association between the risk of *de novo* device-detected atrial fibrillation (AF), the autonomic perturbations before the onset of paroxysmal AF and a 7-days heart rate variability (7dHRV) 1 month after ICD implantation.

**Methods:**

The registry consists of 234 patients implanted with an ICD, including 10 with *de novo* long-lasting atrial tachyarrhythmias with no prior history of AF. The patients were matched via the propensity-score methodology as well as for properties directly influencing the ECGs recorded using GE CardioMem CM 3000. Heart rate variability (HRV) analysis was performed using standard parameters from time- and frequency-domains, and from non-linear dynamics.

**Results:**

No linear HRV was associated with an increased risk of AF (*p* = n.s.). The only significant approach was derived from symbolic dynamics with the parameter “forbidden words” which distinguished both groups on all 7 days of measurements (*p* < 0.05), thereby quantifying the heart rate complexity (HRC) as drastically lower in the *de novo* AF group.

**Conclusion:**

Cardiac autonomic dysfunction denoted by low HRC may be associated with higher AF incidence. For patients with mild to moderate heart failure, standard HRV parameters are not appropriate to quantify cardiac autonomic perturbations before the onset of AF. Further studies are needed to determine the individual risk for AF that would enable interventions to restore autonomic balance in the general population.

## Introduction

Atrial fibrillation (AF), the most common clinical arrhythmia, is associated with increased risk of stroke, heart failure (HF) and possibly dementia (Mozaffarian et al., 2016). There is growing evidence that cardiovascular disease (CVD) risk factors and CVD itself explains only 50% of AF occurrences (Agarwal et al., 2017), as the understanding of AF pathophysiology is still incompletely understood (Benjamin et al., 2009). Cardiac autonomic dysfunction has long been suspected in the development of AF (Coumel, 1994), therefore the direct observation of heart rate variability (HRV) immediately preceding the onset of arrhythmia will likely permit the documentation of the underlying mechanism(s). However, the role of the autonomic nervous system should be taken into consideration for optimal individual antiarrhythmic treatment.

Twenty years ago, the Task Force of the European Society of Cardiology (ESC) and the North American Society of Pacing and Electrophysiology published the HRV standards of measurement (Malik et al., 1996). The contemporary literature has a plethora of studies applying HRV methodologies and their successes in clinical applications. However, the identification of high-risk patients has been rather limited. Recently, the e-Cardiology ESC Working Group and the European Heart Rhythm Association co-endorsed by the Asia Pacific Heart Rhythm Society wrote a joint position statement about advances in HRV signal analysis (Sassi et al., 2015). They presented a critical review of newly developed HRV methodologies developed after publication of the initial Task Force HRV overview (Wessel et al., 2016) and their applications in different physiological and clinical studies.

In a letter (Wessel et al., 2016) in response to this review, we provided several potential explanations for the limited success that merits additional attention: (1) the importance of monitoring respiration for the interpretation of standard HRV analysis, (2) the need to address its complexities using improved signal processing method (Benjamin et al., 2009; Wessel et al., 2009, 2016; Sidorenko et al., 2016) for patients with mild to moderate HF, standard HRV parameters may not be suited due to the alternating sinus rhythm phenomena (Voss et al., 2006; Costa et al., 2017). Therefore, non-linear methods for the description of heart rate complexity may be more appropriate (Kurths et al., 1995; Voss et al., 1996; Wessel et al., 2000; Brindle et al., 2016).

To capture the parameters from these domains, it is necessary to focus on their physiological interpretation. Spontaneous fluctuations of cardiovascular signals were already described more than a 100 years ago (Ludwig, 1847; Koepchen and Thurau, 1959; Wolf et al., 1978) with fluctuations in both heart rate and blood pressure representing oscillations around fixed values and expressing several influences, e.g., respiration and different self-regulating rhythms.

Short-term heart rate regulation is mainly accomplished by neural sympathetic- and parasympathetic-mediated cardiac baroreflexes and peripheral vessel resistance whereas long-term regulation is achieved by hormonal pathways as well as other systems like the renin-angiotensin-system (Berntson et al., 1997). HRV measurements have proven to be independent predictors of sudden cardiac death after acute myocardial infarction, chronic HF or dilated cardiomyopathy (Kleiger et al., 1987; Malik et al., 1996; Tsuji et al., 1996; Szabó et al., 1997). Moreover, it has been shown that short-term HRV analysis yields a prognostic value in risk stratification independent of clinical and functional variables (La Rovere et al., 2003). However, the underlying regulatory mechanisms are still poorly understood. Further work includes the relevance of premature ventricular ectopic beats, which are associated with an increased risk of sudden cardiac death, as well as their sophisticated neuro-cardio-respiratory interactions (Schmidt et al., 1999; Schulte-Frohlinde et al., 2001, 2002), and different means to analyze HRV as stationary epochs (Bernaola-Galván et al., 2001; Camargo et al., 2014).

The main objective of the REACT DX registry was to evaluate different standard therapies to episodes of long-lasting device-detected atrial tachyarrhythmias (duration >6 min at a frequency >190 beats per minute) and assess the quality of sensing and stability of the lead and implantable cardioverter-defibrillator (ICD) (BIOTRONIK Lumax VR-T DX and successor) over the pre-defined 1-year follow-up period. In addition, cardiac autonomic perturbations before the occurrence of *de novo* AF were investigated. Therefore, the 7-day continuous ECGs recordings 1 month after ICD implantation were analyzed in all patients. Based on standard time- and frequency-domain parameters as well as from non-linear dynamics (Wessel et al., 2007), the study also analyzed the association between the risk of *de novo* AF and 7-day HRV (7dHRV).

## Materials and Methods

### Cohort

Between November 2012 and June 2016, the multicentre REACT DX registry enrolled 234 patients before or ≤1 month after ICD implantation from 14 sites in Germany. Follow-up duration was at least 12 months and up to 24 months (18.8 ± 4.9 months).

Of these, 199 (85%) were male and 180 (77%) received the ICD for the primary prevention of sudden cardiac death. The mean (±standard deviation) left ventricular ejection fraction was 33.6 ± 12.8%, 154 (65.8%) had conorary artery disease, 78 (33.3%) had dilated cardiomyopathy and 172 (73.5%) had hypertension. Pharmacological HF therapy was optimized prior to study inclusion in all patients resulting in 218 (93.2%) patients receiving β-blockers, 196 (83.8%) angiotensin ± neprilysin inhibitors, 112 (47.9%) spironolactone, 172 (73.5%) diuretics and 151 (64.5%) statins. During follow-up, a total of 14 (6.0%) patients developed long-lasting atrial tachyarrhythmias (≥6 min). For 10 of these 14 patients, all clinical data as well as the 7-day long-term ECG were available, and they were defined as our *de novo* AF group (first AF occurrence 211 ± 120 day). From the 166 out of the 234 patients with primary prevention and without detected long-lasting atrial tachyarrhythmias, a control group of equal size was created.

The main inclusion criteria were (i) indications and contraindications for ICD implantation according to national and international guidelines, (ii) be available for follow-up visits on a regular basis at an approved investigational centre, (iii) have a first implantation of an ICD-system, (iv) sign the informed consent form, (v) implantation of a Lumax 740 VR-T DX or successor and (vi) have sufficient coverage of mobile phone network.

The main exclusion criteria were (i) presence of permanent atrial tachyarrhythmia, (ii) indication for cardiac resynchronization therapy, (iii) life expectancy of less than 6 months, (iv) expected cardiac surgery within 6 months after enrolment, (v) age less than 18 years and (vi) enrolment in another cardiac clinical investigation.

Device-detected episodes of atrial tachyarrhythmia were evaluated by the investigators and by a clinical event committee consisting of two experienced physicians.

The REACT DX registry was approved by the ethic committee. All patients signed informed consent. The registry was conducted according to GCP and the Declaration of Helsinki, and was registered in Deutschen Register Klinischer Studien (registration number DRKS00010898).

### Pre-processing

The analysis of HRV is often difficult due to many artifacts of the arrhythmias signals. While occasional ectopic beats are treated successfully by most pre-processing methods, more complex arrhythmias or arrhythmias which are similar to normal fluctuations, may remain undetected. We therefore developed a method for data pre-processing which is described in detail elsewhere (Wessel et al., 2007).

A detailed motivation, physiological introduction and the purpose of the analysis of HRV analysis can be reviewed in the Task Force HRV (Malik et al., 1996). It is important to address each artifact with the appropriate tools without influencing the results and the simple exclusion of ventricular premature beats may lead to erroneous HRV parameters (Lippman et al., 1994).

An appropriate method to handle most of these problems is through adaptive filtering, which has been described in detail elsewhere (Wessel et al., 2007). The main advantage of this method is the spontaneous adaptation to variability changes, which enables a more reliable removal of artifacts, ventricular premature beats and VPCs. This filtering algorithm consists of three sub-procedures:

(i) the removal of obvious recognition errors
(ii) the adaptive percent-filter
(iii) the adaptive controlling filter

It must be mentioned our group has analyzed several thousand human and animal time series (Wessel et al., 2007). In those analyses, small variations of the controlling parameters did not significantly influence the filtering results. A MATLAB implementation of the pre-processing algorithm is available from tocsy.agnld.uni-potsdam.de.

### Time- and Frequency-Domain Parameters

Standard methods of HRV analysis include time- and frequency-domain parameters that are linear methods. Time-domain parameters are based on simple statistical methods derived from the RR-intervals as well as the differences between them. Mean heart rate is the simplest parameter, but the standard deviation over the entire time series (sdNN) is the most prominent HRV measure for estimating overall HRV. A list of selected parameters with a short explanation is given in Table A1 and has been described before (Wessel et al., 2007).

### Non-linear Analysis of Heart Rate Variability

Heart rate and blood pressure variability reflects the complex interactions of many different controlled loops of the cardiovascular system. In relation to the complexity of the sinus node activity modulation system, a predominantly non-linear behavior has to be assumed. Thus, the detailed description and classification of dynamic changes using time and frequency measures is often not sufficient. We have previously shown that symbolic dynamic is an efficient approach to analyze the dynamic aspects of HRV (Kurths et al., 1995; Voss et al., 1996). The first step in this analysis is the transformation of the time series into symbol sequences, with symbols given an alphabet letter. Although some detailed information is lost in this process, the broad dynamic behavior can be analyzed (Engbert et al., 1997). Wackerbauer et al. (1994) used the methodology of symbolic dynamics for the analysis of the logistic map where a generic partition is known. However, for physiological time series analysis, a more pragmatic approach is necessary. The transformations into symbols should be chosen in a context-dependent manner. For this reason, we have developed a series of complexity measures based on such context-dependent transformations which have a close connection to physiological phenomena and are relatively easy to interpret (cf. appendix A).

All statistical analyses were performed using the R System. In the case of missing data values, a decision was made on a case to case basis as to if imputation of the missing data was possible or if the variable should be removed. A thorough verification of all patient data was conducted.

### Matching

To investigate autonomic perturbations visible through HRV measures, we matched a control group from those that did not register an AF episode during the follow-up period on two levels. To reduce the possible number of subjects, the first matching was based on criteria directly impacting the morphology and interpretation of the ECGs (Table 1). This specific matching was based on the propensity-score methodology (Imbens and Rubin, 2015) with the score used to represent the propensity of the patient to suffer an AF episode before the end of the follow-up period given the demographic and medical data known at enrolment. This matching was meant to account for any previously available information regarding outcomes. Any differences found during the subsequent analysis presented additional and previously unavailable information.

**TABLE 1:** Parameters available for use in matching via the propensity score methodology: Parameters indicated as: “Fixed matching category” are identical for each matched pair, “Allowed for propensity score” were included into the regression to calculate the propensity score which was used to create the match, “Completeness” indicates the availability of this parameter for the cohort (Checkmarks/crosses highlight accepted/rejected parameters).

**Parameter**	**Fixed matching category**	**Allowed for propensity score**	**Propensity score model coefficients**	**Completeness**	**Missing value process**
Age	×	✓	×	100%	×
Gender	×	✓	Female: 0.27	100%	×
BMI	×	✓	−0.009	95%	Imputation
History of atrial fibrillation	×	×	×	100%	×
History of aflut/atrial tachycardias	×	×	×	100%	×
History of SVTs	×	×	×	100%	×
NYHA	×	✓	C: −0.33	75%	Imputation
CHA*2*DS*2*-VASc	×	✓	C: 1.22, ^5: 0.91, ^6: −0.20	100%	×
Effective anticoagulation	×	×	×	100%	×
Diagnosed coronary artery disease	✓	✓	×	100%	×
Diagnosed myocardial infarction	✓	✓	×	100%	×
Coronary artery bypass graft	×	✓	TRUE: 0.06	100%	×
Percutaneous coronary intervention	×	✓	×	100%	×
Dilative cardiomyopathy	✓	✓	×	100%	×
Hypertrophic obstructive cardiomyopathy	✓	✓	TRUE: 0.29	100%	×
Hypertension	✓	✓	×	100%	×
(Inherited) arrhythmogenic diseases	✓	✓	×	100%	×
Short QT-syndrome	×	×	×	6%	Removal
Long QT-syndrome	✓	✓	×	100%	×
Arrhythmogenic right ventricular Cardiomyopathy	✓	✓	TRUE: 1.27	100%	×
Other arrhythmogenic diseases	✓	×	×	100%	×
Diabetes mellitus	×	✓	×	100%	×
Diabetes mellitus with insulin therapy	×	✓	×	100%	×
Valvular disease	×	✓	TRUE: 0.27	100%	×
Renal insufficiency	×	✓	TRUE: −0.31	100%	×
Drugs: Amiodarone	×	✓	×	100%	×
Drugs: Sotalol	×	✓	×	100%	×
Drugs: Flecainide	×	×	×	100%	×
Drugs: Other anti-arrhythmic drug(s)	×	×	×	100%	×
Drugs: Beta blocker	×	✓	×	100%	×
Drugs: ACE-I/AT1-B	×	✓	TRUE: 0.10	100%	×
Drugs: Spironolactone	×	✓	×	100%	×
Drugs: Diuretics	×	✓	×	100%	×
Drugs: Statins	×	✓	TRUE: 0.16	100%	×
Drugs: Other cardiac drugs	×	✓	×	100%	×

### Propensity Score

A linear logistic regression model was selected to separate those who suffered from AF episodes before implantation and those who did not until at least after the follow-up period. This model only used demographic data and variables from the medical history captured during enrolment (variables implying previous AFs were excluded, Table 1). The remaining variables were verified for completeness and, in the case of missing values, they were included using a k-nearest neighbor approach (Templ et al., 2011). The variables that contained more than 25% missing values were excluded. All ordinal factors were translated to orthogonal polynomial contrasts (Chambers and Hastie, 1991) to allow the incorporation of the order relationship into the model. We performed repeated runs of glmnet cross-validation (Friedman et al., 2010) [α = 0.5, each using a rose resampled (Menardi and Torelli, 2014) variant of the data to account for imbalances] to select the appropriate λ. Finally, we fitted the model to the original, but weighted according to the outcome variable imbalance, dataset at the calculated λ. The selected variables, as used in the model and their coefficients, are presented in Table 1. The response value of this model is the propensity score used for the secondary matching process, the matching results are presented in Table 2.

**TABLE 2:** Clinical patient characteristics of the control, as well as the *de novo* AF group.

**Parameter**	**Quantity**	**Controls (*n* = 10)**	***De Novo* AF (*n* = 10)**	***p***
Age	Mean (SD)	65.0 (10.4)	61.3 (10.2)	n.s.
Gender	Male	10	9	n.s.
BMI	Mean (SD)	27.9 (6.0)	25.4 (3.6)	n.s.
No history of atrial fibrillation		10	10	n.s.
No history of aflut/atrial tachycardias		10	10	n.s.
No history of SVTs		10	9	n.s.
LVEF at enrollment	Mean (SD)	35.0 (10.8)	28.5 (13.7)	n.s.
NYHA	I	0	1	n.s.
	II	4	4	
	III	3	2	
	IV	0	0	
CHA*2*DS*2*-VASc	Mean (SD)	2.6 (1.6)	2.4 (1.5)	n.s.
Diagnosed coronary artery disease		5	5	n.s.
Dilative cardiomyopathy		3 (42.9)	4 (57.1)	n.s.
Hypertension		8	8	n.s.
Diabetes mellitus		4	4	n.s.
Amiodarone		2	0	n.s.
Beta blocker		9	10	n.s.
ACE-I/AT1-B		9	9	n.s.
Diuretics		5	7	n.s.
Statins		4	5	n.s.

All long-term ECGs were recorded for 7 days, 1 month after ICD implantation using GE Healthcare CardioMem CM 3000 devices. HRV analyses were performed separately for each of the 7 days. All HRV parameters described above were calculated on a 24-h basis as well as from windowed analyses. For the latter, the time- and frequency-domain parameters were calculated from successive 5-min windows and then averaged over 24 h. The window length for the non-linear HRV parameter was 30 min (Wessel et al., 2007).

## Results

The results presented in Table 3 were derived from the windowed analysis. No HRV parameter from time- and frequency-domain showed a consistent statistically significant differences between the *de novo* AF and the control group for all 7 days (*p* = n.s.). The only successful approach was based on symbolic dynamics. “Polvar10” showed significant differences on day 1 and 2 whereas the Shannon entropy H_*k*_ was significant on 5 days. The parameter “forbidden words,” however, significantly distinguished both groups on all 7 days of measurement as performed independently (*p* < 0.05; the probability for false statistical results is less than 1 to a billion: <0.05^7). This measure quantifies the heart rate complexity which is drastically lower in the *de novo* AF group. The averaged effects size over all 7 days of “forbidden words” is almost one, values greater than 0.8 are interpreted as strong effects. I.e., the mean values “forbidden words” for the *de novo* AF and the control group differ about one standard deviation. To emphasize the strong effect between *de novo* AF and the control groups, we performed the following Monte-Carlo experiment. From each subject we randomly chose one measurement (1 day out of 7) and compared the “forbidden words” values between both groups. Altogether we have seven one day measurements from 20 subjects (10 *de novo* AF + 10 controls), which offer 7^20^ possibilities for such a random selection. To quantify the effect size, we performed a leave one out classification, i.e., a logistic regression model was trained on 19 subjects and the tested while one of the subjects was left out. This leave one out classification was performed 10000 times. In this way we got an overall accuracy of 0.72 (95% CI: 0.71–0.73, *p* < 0.001), a sensitivity of 0.64, a specificity of 0.8, a positive predictive value of 0.76 and a negative predictive value of 0.70. All values differ significantly high from a mere random effect.

**TABLE 3:** Standard time- and frequency-domain parameters as well as non-linear dynamics for all 7 days (means ± standard deviations are given only for day 1).

**Parameter**	**AF day 1**	**CON day 1**	**Day 1**	**Day 2**	**Day 3**	**Day 4**	**Day 5**	**Day 6**	**Day 7**
meanNN	801.3 ± 113.1	891.2 ± 80	0.060	0.063	0.143	0.346	0.312	0.242	0.189
sdNN	33.6 ± 20.3	46.7 ± 14.2	0.119	0.217	0.162	0.288	0.303	0.663	0.177
Rmssd	18.2 ± 12.2	24.3 ± 9.8	0.246	0.530	0.366	0.735	0.670	0.824	0.471
pNN50	0.04 ± 0.05	0.07 ± 0.06	0.326	0.773	0.479	0.994	0.949	0.829	0.713
Shannon	1.58 ± 0.6	1.96 ± 0.34	0.101	0.123	0.089	0.214	0.202	0.295	0.212
Renyi025	1.88 ± 0.58	2.26 ± 0.32	0.087	0.117	0.083	0.224	0.218	0.300	0.178
Renyi4	1.28 ± 0.56	1.62 ± 0.33	0.119	0.140	0.107	0.212	0.210	0.290	0.245
Renyi2	1.41 ± 0.59	1.77 ± 0.34	0.112	0.132	0.098	0.211	0.204	0.294	0.232
pNNl10	0.65 ± 0.25	0.49 ± 0.16	0.118	0.130	0.184	0.346	0.239	0.440	0.296
pNNl20	0.79 ± 0.18	0.69 ± 0.15	0.232	0.295	0.368	0.665	0.426	0.674	0.401
pNNl30	0.9 ± 0.11	0.84 ± 0.11	0.274	0.521	0.488	0.880	0.676	0.918	0.553
P	731.6 ± 706.2	1236.4 ± 694.3	0.135	0.310	0.316	0.340	0.400	0.918	0.212
ULF	75.5 ± 59.3	165.8 ± 82.5	**0.015**	0.177	0.127	0.076	0.187	0.591	0.104
VLF	415 ± 432	801.7 ± 498.6	0.090	0.235	0.281	0.255	0.343	0.623	0.233
LF	190 ± 210	202.8 ± 133.6	0.875	0.721	0.690	0.980	0.791	0.515	0.448
HF	51 ± 59	66.1 ± 36.9	0.508	0.858	0.551	0.906	0.993	0.467	0.302
LF/HF	4.9 ± 2.7	4.5 ± 2.3	0.756	0.508	0.456	0.593	0.943	0.995	0.177
LFn	0.7 ± 0.2	0.7 ± 0.1	0.915	0.508	0.360	0.582	0.676	0.666	0.356
FORBWORD	8.6 ± 9.4	2.2 ± 3.1	**0.049**	**0.039**	**0.046**	**0.031**	**0.049**	**0.033**	**0.036**
H_*k*_	3.4 ± 0.5	3.7 ± 0.2	**0.042**	**0.045**	0.092	**0.048**	**0.049**	**0.034**	0.078
Polvar10	0.4 ± 0.3	0.1 ± 0.1	**0.042**	**0.046**	0.077	0.122	0.102	0.217	0.110

*The results of the Mann–Whitney-*U*-test are presented in the columns Day 1 to 7. No time- and frequency-domain parameters demonstrated consistent significant differences between the *de novo* AF and the control group. In contrast, the symbolic dynamics parameter Forbword and H_*k*_ demonstrated significant differences for several days (Bold font: *p* < 0.05).*

**FIGURE 1:**
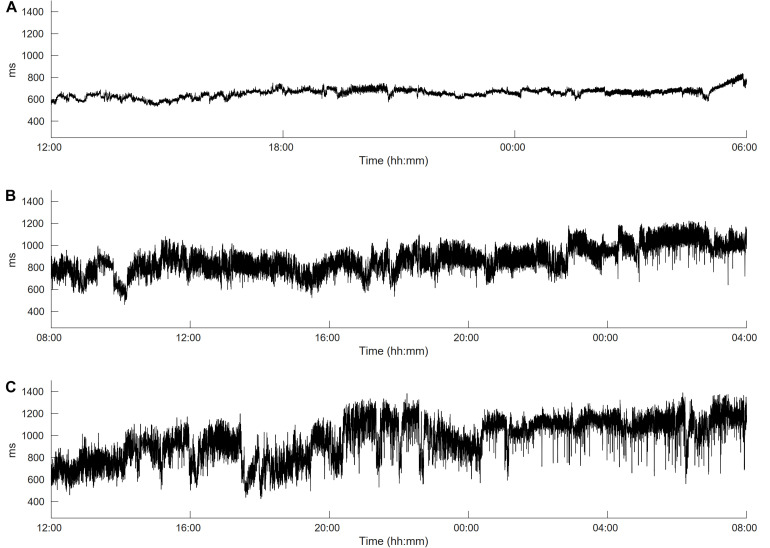
Heart rate variability (HRV) in long-term ECG recordings. **(A)** Time series is from a *de novo* AF patient with a high sympathetic tone. Heart rate is strongly increased (86 bpm) and HRV is clearly decreased (day time sdNN = 12 ms), **(B)** time series is also from a *de novo* AF patient but with normal heart rate and HRV (65 bpm, day time sdNN = 98 ms) and **(C)** time series is from a control patient with normal heart rate and HRV (64 bpm, day time sdNN = 105 ms).

**FIGURE 2:**
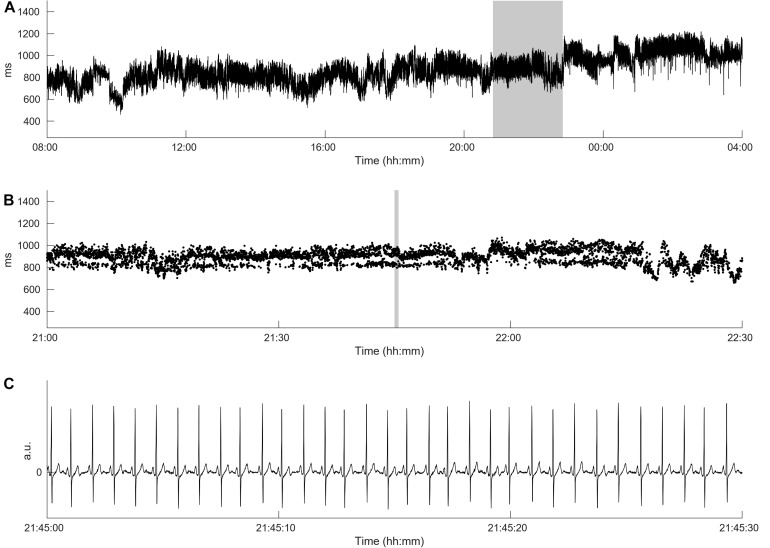
Heart rate complexity of the *de novo* AF patient with normal heart rate and HRV from Figure 1B. Panel **(A)** shows the original time series from Figure 1 demonstrating a high HRV over the day. Panel **(B)** presents a time breakdown of the gray area from panel **(A)** and reveals the occurrence of alternating sinus rhythm patterns. The HRC is strongly decreased, therefore FORBWORD is increased to 8 which is comparable to the HRC value presented in Figure 1A. Panel **(C)** shows the ECG from the gray area from panel **(B)** showing sinus rhythm was maintained, (cf. Costa et al., 2017).

**FIGURE 3:**
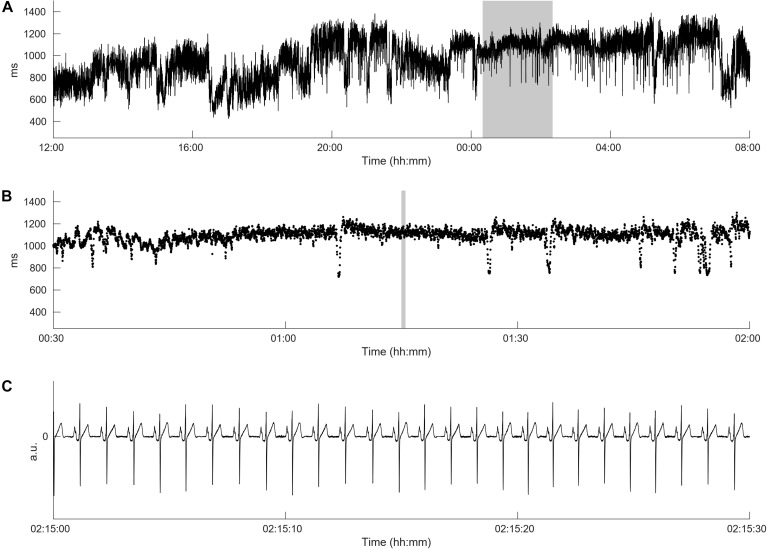
Heart rate complexity of the control patient with normal heart rate and HRV from Figure 1C. Panel **(A)** shows the original time series from Figure 1 demonstrating a high HRV over the day. Panel **(B)** present a time breakdown from the gray area from panel **(A)**. No alternating sinus rhythm patterns can be detected. The HRC is not decreased, therefore FORBWORD is equal to 1. Panel **(C)** shows the ECG from the gray area from panel **(B)** demonstrating sinus rhythm.

Figure 1 demonstrates the differences between HRV in long-term ECG recordings. It is well known that cardiac autonomic dysfunction, characterized by a high sympathetic tone as observed in Figure 1A and documented via an increased heart rate, is associated with a strongly decreased HRV and a strongly increased risk of AF occurrence (Jons et al., 2010; Nortamo et al., 2018). In this case, not only the HRV but also the heart rate complexity is low (FORBWORD = 12). Figures 1B, 2 show one example of a *de novo* AF patient with “normal” heart rate and HRV. A decrease in complexity is not readily visible at first glance, however, heart rate complexity is decreased due to the occurrences of alternating sinus rhythm patterns (Costa et al., 2017). The time series in Figures 1C, 3 are from a control patient with normal heart rate, HRV and heart rate complexity. The patterns of the two other patients are undistinguishable using only standard HRV parameters. Only non-linear parameters are able to show a strong decrease in HRC and an increase in FORBWORD due to alternating sinus rhythm patterns present in the times series of Figure 2, which are unremarkable for the time series in Figure 3.

## Discussion

The objective of this investigation was to study the association between the risk of *de novo* AF and 7dHRV based of standard time- and frequency-domain parameters as well as from non-linear dynamics. Therefore, the long-term ECGs of 20 of the 234 patients with mild to moderate HF included in the REACT DX registry were analyzed. For all patients, the implantation of an ICD device was indicated suggesting all were high-risk patients. The main aim of the present study was to detect the patients with a high risk for *de novo* AF based on HRV parameters. This was a complex process for several reasons: (i) we did not have the simple task of separating patients from a healthy group as done previously (Costa et al., 2017), (ii) a high amount of ectopy was expected, (iii) patients often had high heart rates and low HRV, (iv) our patient match included different disease states, (v) we did not exclude inflammation and fibrosis, (vi) patient were prescribed different medication regimens and (vii) patients may have had different circadian HRV profiles. Therefore, standard HRV parameters failed to predict cardiac autonomic dysfunction and no parameter from time- and frequency-domain showed significant differences between the *de novo* AF and the control groups. In contrast, the only significant approach was derived from symbolic dynamics with the parameter “forbidden words” which distinguished both groups on all 7 days of measurements independently (*p* < 0.05), thereby quantifying the HRC as drastically lower in the *de novo* AF group.

It is known that cardiac autonomic dysfunction, as evidenced by severely diminished HRV, increases the risk of AF occurrence (Jons et al., 2010). However, these perturbations may not be detected by standard HRV analysis, but appear to be a marker of higher AF incidence (Table 2 and Figure 1). A decreased in heart rate complexity may be accompanied with a decreased in HRV. Researchers (Costa et al., 2017) recently referred to the alternating sinus rhythm pattern phenomenon as “heart rate fragmentation.” Applying standard HRV methods to such time series with alternating rhythms may have lead to false or incomplete interpretations as reported by (Bettoni and Zimmermann, 2002) who found a vagal predominance before the onset of AF, and (Vikman et al., 1999) who detected an altered complexity. Others (Costa et al., 2017) also reported abnormal patterns in Poincaré plots and other maps (Woo et al., 1992; Brouwer et al., 1996; Huikuri et al., 1996; Domitrovich and Stein, 2003; Stein et al., 2005, 2008; Gladuli et al., 2011; Makowiec et al., 2015). The pathophysiology of alternating sinus rhythm patterns remains to be determined. Different mechanisms may be involved such as the sinus node exit block, a very subtle atrial bigeminy and the sinus node parasystole and perturbations of internal pacemaker “clocks” in the SA node (Costa et al., 2017). Costa et al. (2017) speculated that heart rate fragmentation would be of high interest if it was an initial event leading to arrhythmias such as AF or other tachyarrhythmias. The results of our study confirmed this hypothesis.

Costa et al. (2017) raised the additional question whether abnormalities in breathing dynamics could be responsible for the fragmentation. An indirect indication of cardiorespiratory coupling involvement is that deep breathing may lead to alternating patterns in heart rate (Löwit, 1879). Following earlier work on cardio-respiratory synchronization changes with sleep and aging (Bartsch et al., 2012), we (Riedl et al., 2014) introduced the analysis of cardiorespiratory coordination (CRC) during sleep. They found, by using the advanced analysis technique of the coordigram, not only the occurrence of CRC was significantly more frequent during respiratory sleep disturbances than during normal respiration but also more frequent after these events. A further investigation of CRC (Krause et al., 2017) showed the new phenomenon of heartbeat-initiated inspiration at the end of an apnea, which provides new impulses to current approaches to obstructive sleep apnea characterization (Ivanov et al., 1996; Mietus et al., 2000).

The major limitation of our study is the low number of *de novo* AF events as only 10 patients were included. AF is a powerful risk factor for stroke, independently increasing risk about five-fold throughout all age groups (Mozaffarian et al., 2016). Because AF is often asymptomatic and likely frequently undetected, the risk of stroke attributed to AF may be substantially underestimated. In the setting of AF, important risk factors for stroke include advancing age, hypertension, HF, diabetes mellitus, previous stroke or TIA, vascular disease and female sex (Schmitt et al., 2009; Mozaffarian et al., 2016). Additional biomarkers such as high levels of troponin and BNP also increase the risk of stroke independent of the well-established clinical characteristics (Mozaffarian et al., 2016). Thus, further studies are needed to determine the individual risk for AF based on HRV, heart rate complexity, clinical characteristics and additional biomarkers.

## Conclusion

Cardiac autonomic dysfunction denoted by low heart rate complexity may be associated with a higher AF incidence. For patients with mild to moderate HF, standard HRV parameters are not appropriate to quantify cardiac autonomic perturbations before the first occurrence of AF. Further studies are needed to determine the individual risk for AF that would enable interventions to restore autonomic balance in the general population.

## Disclosure

CK has received lecture honorary/travel support from Abbott Medical (St. Jude Medical), BIOTRONIK, Boston Scientific, Bristol Myers Squibb, LivaNova, Medtronic, Novartis, Phillips (Spectranetics), he is/has recently been advisor to BIOTRONIK and LivaNova and performs/has recently performed clinical studies supported by Abbott Medical (St. Jude Medical), BIOTRONIK, Boston Scientific and LivaNova. NW, KB, and JFK received support from BIOTRONIK.

## Data Availability Statement

The raw data supporting the conclusions of this article will be made available by the authors, without undue reservation.

## Ethics Statement

The studies involving human participants were reviewed and approved by DRKS00010898. The patients/participants provided their written informed consent to participate in this study.

## Author Contributions

NW, TH, RB, and AS designed and directed the project. TH, KR, RB, AS, DW, SK, CK, and NK performed the register. NW, AG, KB, JFK, and JK analyzed the data. NW, JK, KB, AG, and KR wrote the manuscript. All authors contributed to the article and approved the submitted version.

## Conflict of Interest

The authors declare that the research was conducted in the absence of any commercial or financial relationships that could be construed as a potential conflict of interest.

## Appendix A

### Symbolic Dynamics

While comparing different types of symbol transformations, we found that the use of four symbols, as explained in Eq. (1), was appropriate for our analysis. The time series x_1_, x_2_, x_3_…, x_*N*_ is transformed into the symbol sequence s_1_, s_2_, s_3_…, s_*N*_, s_*i*_ ∈ A on the basis of the alphabet A = {0,1,2,3}.

(1) $$s_{i}\,\left(x_{i}\right)=\left\{ \begin{array}{c} 0:\mu <x_{i}\leq \left(1+a\right)\,\mu \\ 1:\left(1+a\right)\,\mu <x_{i}\leq \infty \\ 2:\left(1-a\right)\,\mu <x_{i}\leq \mu \\ 3:0<x_{i}\leq \left(1-a\right)\,\mu \end{array}\right.\;\mathrm{where}\,i=1,2,3,\ldots$$

The transformation into symbols refers to three given levels where μ denotes the mean beat-to-beat interval and *a* is a special parameter that we have chosen 0.05. We tested several values of *a* from 0.02 to 0.08, however the resulting symbol sequences differed not significantly (Wessel et al., 2007). Because there are several quantities that characterize such symbol strings, we analyzed the frequency distribution of words of length 3 (i.e., substrings which consist of three adjacent symbols leading to a maximum of 64 different words), which is a compromise between retaining important dynamical information and having a robust statistical estimate of the probability distribution.

We considered 3 measures of complexity:

(i) The Shannon entropy *H*_*k*_ calculated from the distribution *p* of words is the classic measure for the complexity in time series:

(2) $$H_{k}=-\sum_{\omega \,e\,W^{k}\cdot p\,\left(\omega \right)>0}p\,\left(\omega \right)\,\log p\,\left(\omega \right)$$

where *W*^*k*^ is the set of all words of length *k*. Larger values of Shannon entropy refer to higher complexity in the corresponding tachograms and lower values to lower ones.
(ii) The “forbidden words” in the distribution of words of length 3 which are the number of words that (almost) never occur (with probability of less than 0.1%). A high number of forbidden words reflects a rather regular behavior in the time series and, if the time series is highly complex in the Shannonian sense, only a few forbidden words are to be found.
(iii) The parameter “plvar10” characterizing short phases of low variability from successive symbols of another simplified alphabet *B* and consisting only of symbols “0” and “1.” In our study, “0” stands for a small difference between two successive RR-intervals (the resolution of the defibrillators) whereas “1” represents cases when the difference between two successive RR-intervals exceeds a certain specific limit.

$$s_{n}=\left\{ \begin{array}{c} 1:\left|x_{n}-x_{n-1}\right|\geq 10\,\text{msec}\\ 0:\left|x_{n}-x_{n-1}\right|<10\,\mathrm{msec}. \end{array}\right.$$

Words consisting of a unique type of symbols (either all “0” or all “1”) were counted. To obtain a statistically robust estimate of the word distribution, we chose words of length six, defining a maximum of 64 different words. “Plvar10” represents the probability of the word “000000” occurrence and thus detects even intermittently decreased HRV.

**TABLE A1:** Description of the selected time- and frequency-domain parameters, standard and additional measures (Wessel et al., 2007).

**Variable**	**Units**	**Definition**
meanNN	[ms]	Mean BBI
sdNN	[ms]	Standard deviation of all BBI values
Rmssd	[ms]	Root mean square of successive BBI differences
pNN50	%	Percentage of NN-interval differences greater than 50 ms
pNNlX	%	Percentage of beat-to-beat differences lower than X ms (e.g., *X* = 10/20/30 ms)
Shannon	None	Shannon entropy of the histogram (density distribution of the BBIs)
RenyiX	None	Renyi entropy of order X of the histogram (e.g., *X* = 2/4/0.25)
P	ms^2^	Total power from 0 to 0.4 Hz
ULF	ms^2^	Ultra-low frequency band 0–0.0033 Hz
VLF	ms^2^	Very low frequency band 0.0033–0.04 Hz
LF	ms^2^	Low frequency band 0.04–0.15 Hz
HF	ms^2^	High frequency band 0.15–0.40 Hz
LF/HF	None	Quotient of LF and HF
LFn	None	Normalized low 0 frequency band (LF/(LF + HF))

*BBI stands for the filtered beat-to-beat intervals (NN-intervals).*
